# A Guide to the Generation of a 6-Hydroxydopamine Mouse Model of Parkinson’s Disease for the Study of Non-Motor Symptoms

**DOI:** 10.3390/biomedicines9060598

**Published:** 2021-05-25

**Authors:** Débora Masini, Carina Plewnia, Maëlle Bertho, Nicolas Scalbert, Vittorio Caggiano, Gilberto Fisone

**Affiliations:** 1 Department of Neuroscience, Karolinska Institutet, 171 77 Stockholm, Sweden; masini@sund.ku.dk (D.M.); carina.plewnia@ki.se (C.P.); maelle.bertho@ki.se (M.B.); nscalbert.carvajal@gmail.com (N.S.); caggiano@gmail.com (V.C.); 2 Department of Neuroscience Faculty of Health and Medical Sciences, University of Copenhagen, Blegdamsvej, 3B, 2200 Copenhagen, Denmark

**Keywords:** Parkinson’s disease, 6-hydroxydopamine, non-motor symptoms, prodromal, mouse, basal ganglia, striatum, bilateral, behavior, rodent surgery

## Abstract

In Parkinson’s disease (PD), a large number of symptoms affecting the peripheral and central nervous system precede, develop in parallel to, the cardinal motor symptoms of the disease. The study of these conditions, which are often refractory to and may even be exacerbated by standard dopamine replacement therapies, relies on the availability of appropriate animal models. Previous work in rodents showed that injection of the neurotoxin 6-hydroxydopamine (6-OHDA) in discrete brain regions reproduces several non-motor comorbidities commonly associated with PD, including cognitive deficits, depression, anxiety, as well as disruption of olfactory discrimination and circadian rhythm. However, the use of 6-OHDA is frequently associated with significant post-surgical mortality. Here, we describe the generation of a mouse model of PD based on bilateral injection of 6-OHDA in the dorsal striatum. We show that the survival rates of males and females subjected to this lesion differ significantly, with a much higher mortality among males, and provide a protocol of enhanced pre- and post-operative care, which nearly eliminates animal loss. We also briefly discuss the utility of this model for the study of non-motor comorbidities of PD.

## 1. Introduction

Parkinson’s disease (PD) is still diagnosed based on the emergence of rest tremor, rigidity and bradykinesia [1]. These motor symptoms develop in response to the progressive loss of dopamine neurons located in the substantia nigra pars compacta (SNc), which project to the dorsal striatum. In line with this traditional view, animal models of PD have been typically generated by targeting the nigrostriatal pathway with toxins [2] or genetic interventions [3,4,5] that reproduce motor deficits. However, the full pathological spectrum of PD extends well beyond that of a hypo-dopaminergic movement disorder, and includes autonomic dysfunctions (e.g., gastrointestinal and urinary dysfunctions, and orthostatic hypotension), sleep and wakefulness disturbances, and a wide range of neuropsychiatric conditions [4,5]. These non-motor symptoms often develop in parallel to the loss of noradrenaline-, serotonin-, and acetylcholine-containing structures, which occurs in concomitance, or even before the loss of dopamine neurons [6]. Whereas the motor symptoms of PD are successfully counteracted by replacement therapies based on L-DOPA and dopamine receptor agonists [7,8], non-motor symptoms are often refractory to these standard approaches [4,5]. In this context, animal models which can replicate at least in part these comorbidities represent a valuable tool to improve the current management of PD.

6-hydroxydopamine (6-OHDA) was identified more than 50 years ago as a catecholamine selective neurotoxin [9,10] and is still widely used to reproduce PD [2,11,12]. The dopamine and noradrenaline transporters mediate the intracellular accumulation of 6-OHDA [13], which leads to neuronal death through a combination of oxidative stress-induced cytotoxicity [14,15] and impaired mitochondrial function [16]. The neurodegenerative response to 6-OHDA is not accompanied by the appearance of Lewy bodies inclusions, which are typically observed in PD. This may be related to the mechanism of action of this toxin and to its rapid effect, which contrasts with the progressive character of PD.

In spite of these limitations, the relative selectivity of 6-OHDA has been at the basis of its popularity to model the classic motor symptoms of PD, as well as the motor complications associated with prolonged administration of standard antiparkinsonian medications, such as L-DOPA-induced dyskinesia. However, 6-OHDA rodent models of PD have also been employed to replicate several non-motor comorbidities frequently observed in patients. For instance, injection of 6-OHDA into the medial forebrain bundle (MFB), SNc, or striatum of mice and rats results in cognitive and affective deficits, accompanied by olfactory impairment and disruption of circadian rhythm and sleep [17,18,19,20]. These abnormalities can be in part explained by the concomitant degeneration of the noradrenaline system [21,22], but also by the reduction in serotonin levels produced by the 6-OHDA lesion [23,24]. Moreover, bilateral injections of 6-OHDA in the rat dorsal striatum have been reported to decrease the number of orexin neurons in the lateral hypothalamus [25], which are involved in the regulation of sleep-wake and respiration, in addition to the control of arousal, motivation and ingestive behavior [26,27,28]. Altogether, these observations indicate that the effects produced by 6-OHDA are not limited to the classical degeneration of the dopamine system, and that models based on this neurotoxin can be successfully employed to study non-motor symptoms.

6-OHDA is commonly injected unilaterally in the SNc, MFB, or in the striatum. Injection in the SNc results in a rapid (12 h) onset of neuronal loss and a more progressive fiber degeneration during the following 7–10 days [29,30]. When injected in the MFB, 6-OHDA causes a near complete loss of dopamine fibers and cell bodies 3 and 5 weeks, respectively, after lesioning [31]. Striatal injection of 6-OHDA depletes most of the local dopamine innervation already 24 h after injection [32]. This initial effect is followed by a reduction in the number of dopamine cell bodies in the substantia nigra, which may progress for up to 3 weeks [32,33,34]. The unilateral injection of 6-OHDA has been extensively used to study motor deficiencies and complications associated with parkinsonism, by assessing rotational behavior and other asymmetrical movements produced spontaneously or in response to anti-parkinsonian drugs [35,36].

The larger size of the striatum, in comparison to SNc or MFB (Supplementary Figure S1a), allows for a better control of the extent of the 6-OHDA lesion, particularly when using mice. Striatal 6-OHDA injections can be designed to produce a partial loss of dopamine, associated with mild motor symptoms, thereby reproducing the early stages of PD [21,37,38]. This approach is well suited to the study of non-motor symptoms, since these ailments often appear during the prodromal phase of PD. Moreover, because of its more restricted effects, the partial 6-OHDA lesion can be more easily performed bilaterally, which simplifies the analysis of non-motor behaviors by eliminating the interference of distorted posture and movement laterality typically observed with unilateral lesions.

In this article, we describe a procedure to generate a mouse model of PD based on a bilateral partial 6-OHDA lesion of the dorsal striatum. This model has been previously shown to replicate non-motor comorbidities, including memory deficits [21,39], depression- and anxiety-like behavior [40,41], impaired olfactory discrimination [40] and disrupted rest-activity circadian rhythm [39] (Table 1). Particular attention is given to reduce mortality, which is frequently observed in 6-OHDA-lesion mice. The protective effect of an enhanced care (EC) protocol based on a combination of pre- and postoperative interventions is compared with that of a standard care (SC) protocol commonly used after stereotaxic surgery. We also provide key information on gender differences in vulnerability and highlight critical factors that influence survival, thereby facilitating animal care and avoiding unnecessary losses.

## 2. Materials and Methods

### 2.1. Animals

Male and female C57Bl/6 mice weighting 22–32 g on surgical day were used throughout the study. Groups of 3–5 animals were housed in individually ventilated cages (GM500; Tecniplast, Italy) under a 12 h light–dark cycle with food (standard chow) and water ad libitum, at 21–22 °C and 50–55% humidity. All procedures were approved by the local ethical committee (Stockholm Norra Djuretiska nämn) and followed the EU directive (2010/63/EU) under the ethical permit 12148-17.

### 2.2. Pre-Operative Care

Mice purchased from external sources were acclimatized to the facility for at least one week prior to any experimental manipulation. This period allowed animals to adjust to the new environment and promoted both animal welfare and reproducible experimental results.

**Figure 1:**
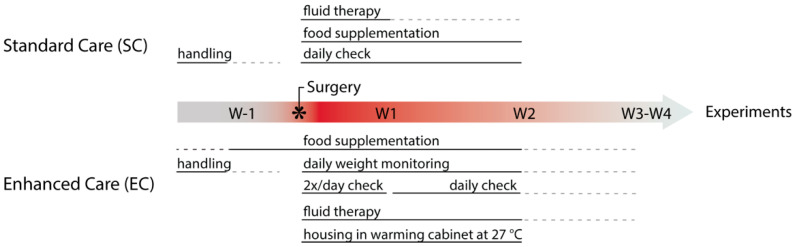
Timeline showing perioperative steps implemented during standard and enhanced care protocols. EC (lower panel) contains additional steps that increase animal welfare (see text for further information). W, week.

Figure 1 illustrates the SC and EC protocols applied in this study. Prior to surgery, all mice were identified (sex, age, health status and weight) and habituated by gentle handling to reduce stress [42,43]. Cage enrichment, such as hiding places and nesting material is implemented to reduce post-operative distress [44]. In EC, supplementary food was given to both sham and lesion mice, starting one week prior to surgery to prevent food neophobia [45]. Thus, mice subjected to EC were introduced to soft wet-chow pellets (standard chow, 3.6 Kcal/g, SDS RM3, Scanbur) combined with one of the following supplementary foods: DietGel Boost (3.69 Kcal/g, Clear H2O, Scanbur), High-Fat Diet (5.24 Kcal/g, Open Source Diets, Taconic) or sweetened condensed milk (diluted 1:1 in water, 1.4 Kcal/g). The supplementary food is palatable, easily accessible and was provided daily in a limited amount (~5 g/mouse, standard chow ad libitum). Note that supplementary food is discontinued upon recovery (normally on week (W) 2 but latest 4 weeks after surgery, W4) before the experiment’s start.

Prior to surgery, fasting should be avoided, since the mouse’s high metabolic rate will quickly deplete energy reserves. Cage changes can lead to increased home-cage activity, with potential weight loss, and should be avoided on the day before surgery.

### 2.3. 6-OHDA Surgery

**Figure 2:**
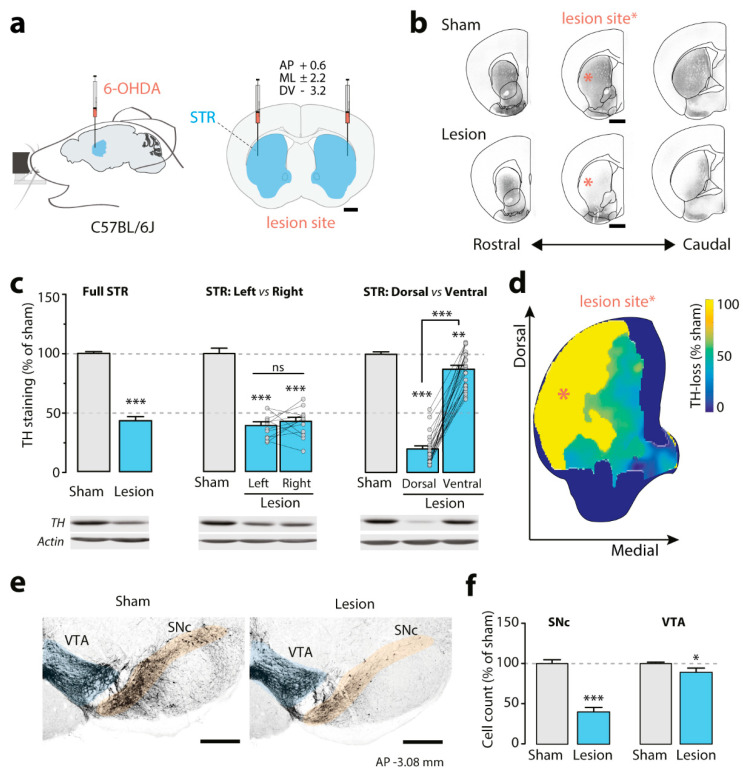
Bilateral partial striatal lesion with 6-OHDA. (**a**) Schematic illustration of the stereotaxic apparatus employed to inject 6-OHDA into the dorso-lateral part of the striatum (STR, blue). The coronal section on the right shows the targeted positions (scale 1 mm). (**b**) Representative coronal sections showing immunolabelling of dopaminergic fibers by TH-antibody in sham and 6-OHDA-lesion mice. Raw images of slices surrounding the injection site are presented with inverted color (scale: 1 mm, position from bregma: 1.42, 0.60 and 0.26 mm, in rostro-caudal order). (**c**) Percentage of TH-loss in 6-OHDA lesion mice compared with sham-lesion mice as quantified by Western blot. Values were normalized to sham group. Left, data obtained from bilateral free-hand dissection of whole striatum (full STR), showing a 56.4 ± 4.73% reduction in TH (*n* = 8 sham and 14 lesion mice; unpaired *t*-test, one-tailed, *p* < 0.0001, t = 11.92, df = 20). Middle, TH levels in whole left and right striata from 6-OHDA lesion mice, in comparison to sham striata (*n* = 8/12/12 per group, in order as shown in the graph; one-way ANOVA, Dunnett’s vs. sham, *p* < 0.0001). Note the similar decrease in left and right striata (left vs. right, ratio paired *t*-test, two tailed, *p* = 0.5267, t = 0.6538, df = 11). Right, comparison between the level of TH immunoreactivity in punches from dorsal and ventral striatum of sham and 6-OHDA lesion mice (*n* = 20/24/24 per group, in order as shown in the graph; one-way ANOVA, Sidak’s sham vs. lesion^dorsal^ *p* < 0.0001; sham vs. lesion^ventral^, *p* = 0.0091; lesion dorsal vs. ventral, *p* < 0.001). Lower panels show representative Western blots of TH immunoreactivity. (**d**) Coronal view of lesion site, showing percentage of TH-fiber loss in STR, as quantified via microscopy and section mapping. Total depletion is shown in yellow (*n* = 5 mice/group). (**e**) Representative images of TH immunolabelling of cells within SNc (orange shade) and VTA (blue shade) of sham and lesion mice (scale 500 µm, −3.08 mm from bregma). Division of the two areas was based on the axon bundle (medial lemniscus) separating SNc and VTA. (**f**) Bar graphs show cell count quantifications as % of sham with 59.5 ± 6.8% reduction in TH-positive cells in SNc (*n* = 4 mice per group/8 sections each, unpaired one-tailed *t*-test, *p* < 0.0001, t = 8.757, df = 6) and 10.8 ± 5.3% reduction in TH-positive cells in VTA (*n* = 4 mice per group/8 sections each, unpaired one-tailed *t*-test, *p* = 0.0434, t = 2.041, df = 6). All data are presented as mean ± SEM. * *p* < 0.05, ** *p* < 0.01, *** *p* < 0.001. 6-OHDA: 6-hydroxydopamine, TH: tyrosine hydroxylase, SNc: substantia nigra compacta, VTA: ventral tegmental area.

A detailed step-by-step description of the surgical procedure including surgical equipment and aseptic technique can be found in Supplementary Data. Briefly, mice were anesthetized with 4% isoflurane and positioned in a stereotaxic frame (Stoelting Europe, Dublin, Ireland) equipped with a heating pad (37 °C) to maintain normothermia. All animals were injected subcutaneously with 0.1 mg/kg of buprenorphine (Temgesic, Apoteket, Stockholm, Sweden) at the start of the surgery, and ophthalmic ointment (Oftagel, Santen, Apoteket, Stockholm, Sweden) was applied to the eyes to prevent corneal drying. The lesion was induced by injecting each striatum with 1 μL of 6-OHDA hydrochloride (Sigma Aldrich, Stockholm, Sweden) at a free base concentration of 4 µg/µL dissolved in 0.9% sterile saline with 0.02 mg/mL of ascorbic acid, according to the following coordinates (mm from bregma): anteroposterior (AP) +0.6; mediolateral (ML) ±2.2 and dorsoventral (DV) −3.2 (Figure 2a). Control mice received a sham lesion, consisting of bilateral injections of 1 μL of vehicle (0.02 mg/mL ascorbic acid in 0.9% sterile saline). The amount of 6-OHDA and the injection volume utilized to reproduce a partial lesion were chosen based on previous work in mice, showing that two injections of 6 µg of 6-OHDA in 2 µL per striatum result in nearly total loss of dopamine innervation [46]. To prevent dehydration, all animals were injected subcutaneously with 10 mL/kg of 5% sterile glucose solution at the end of the surgery.

### 2.4. Post-Operative Care

In both protocols, mice received subcutaneous injections of 0.1 mg/kg of Temgesic every 12 h for 48 (SC) or 72 h (EC) post-surgery. Both SC and EC mice were offered easy access to supplementary food by placing it on the cage floor.

During the recovery weeks following surgery, mice subjected to EC were housed in a warming cabinet ~27 °C (IVC Recovery Unit, Tecniplast, Italy) and checked twice a day throughout the first week of recovery. During recovery, W2 EC mice were checked only once every 24 h and then transferred back to normal housing temperature (22 °C). Moreover, in EC mice were weighed daily (at a fixed time) over a period of two weeks and thereafter weighted every 3–4 days, whereas mice treated with SC were housed in normal vivarium conditions and checked once a day for the first 2 weeks.

6-OHDA lesion mice can suffer from a variety of post-operative complications and a continuous health assessment log should be kept for individual mice (see Table 2). The health status of the mice was assessed during the first 14 days post-surgery and animals, regardless of care protocol, that reached the human endpoint (defined by the ethical permit) were euthanized.

Dehydrated mice received fluid therapy such as lactated Ringer’s solution, sterile saline (0.9%) or glucose (5%) subcutaneously or intraperitoneally at 20 mL/kg, upon need [47]. Male mice can suffer from penile prolapse (paraphimosis) and obstructive uropathy after 6-OHDA surgery [48,49]. Thus, the genitals of male mice of the EC protocol were carefully examined daily for paraphimosis and urethral plugs (Table 2). If paraphimosis was observed, lubrication (Oftagel or mineral oil) was applied, and mice were placed on soft bedding to reduce swelling. Urethral plugs are observed as firm proteinaceous material at the proximal urethra (Supplementary Figure S1f) and lead to obstruction of the urinary tract. To remove the plug, lubrication and local analgesic cream were applied (Xylocain, Aspen, Apoteket, Stockholm, Sweden) and short anesthesia (isoflurane) was induced whenever mice showed distress during the procedure.

### 2.5. Immunohistochemistry and Image Analysis

Striatal lesion mapping (Figure 2b,d) was performed in C57Bl/6 male mice (*n* = 5/group, 3 months of age). Mice were deeply anaesthetized with pentobarbital (60 mg/kg, diluted 1:1 in sterile saline (0.9%) intraperitoneal, Sanofi-Aventis, France) and transcardially perfused with 4% (*w*/*v*) paraformaldehyde (Sigma Aldrich, Stockholm, Sweden) in 0.1 M sodium phosphate buffer (pH 7.5, freshly made), 3 weeks after surgery. The brains were removed, postfixed for 12 h at 4 °C. Coronal sections of 60 µm were cut using a vibratome (Leica VT1000S, Nussloch, Germany), washed in tris-buffered saline (TBS)/0.1% Triton X-100 and incubated for 90 min at room temperature in a blocking solution (0.3% Triton X-100, 10% normal goat serum (Jackson ImmunoResearch, Suffolk, UK) in phosphate-buffered saline). Sections were washed in TBS/0.1% Triton X-100 and incubated for 15 h at 4 °C in the same buffer with anti-tyrosine hydroxylase (TH) monoclonal antibody (1:2000; Millipore, Stockholm, Sweden) and 3% bovine serum albumin (Fisher Scientific, Hampton, NH, USA). Immunoreactivity was revealed using a secondary goat Cy3-coupled antibody (1:2000; Jackson Laboratory, Bar Harbor, ME, USA) for 3 h at room temperature. Sections were then stained with Nissl, washed and mounted in 2.5% DABCO (Sigma Aldrich, Stockholm, Sweden) and kept at 4°C. Images were taken at 10× magnification (24 bit depth, 96 dpi, 12,288 × 9088 pixels) using a fluorescence microscope (Zeiss Axio Imager M1, Jena, Germany) and Neurolucida software. TH example sections presented in Figure 2b were color inverted, transformed into greyscale and adjusted as a batch to increase clarity (ImageJ). The probability image shown in Figure 2d was generated from tissue sections stained for TH, processed, and reconstructed in MATLAB [50]. All striatal sections were serially collected, and every 3rd section (60 µm each) was scanned for analysis and imaged (10×, tile, full section). The analysis consisted of two parts. First, anatomical landmarks were identified based on the Nissl staining. Second, slices were matched (affine transformation followed by cubic B-spline transformation) to the coordinate framework (CCF v3) of the Allen Mouse Brain Atlas at 25 μm resolution with custom-made MATLAB scripts. Projection to the standardized Allen Mouse Brain Atlas was performed via the B-spline maps. The script was developed to facilitate automated whole brain analysis of tissue sections in a manner similar to serial two-photon tomography [51] and light sheet microscopy [52]. Last, z-score staining-intensity values were calculated for each individual mouse (*n* = 5/group) and finally calculated as % of sham mice. The resulting probability image for striatal TH-loss neighboring the injection site was color coded as a heatmap.

Cell count quantification in the SNc and ventral tegmental area (VTA) (Figure 2e,f) was performed in C57Bl/6 mice of both genders (*n* = 4/group, 3 months of age). Animals were transcardially perfused and coronal sections of 40 µm were stained against TH following the above-mentioned protocol. For each animal, 8 alternating sections were selected −2.9 to −3.6 mm from bregma. Tiled images with a pixel size of 0.39 µm^2^ including the SNc and VTA were captured using sequential laser scanning confocal microscopy (Zeiss LSM, Carl Zeiss, Germany) at 10× magnification. Acquired images were transformed into greyscale and TH immunofluorescence quantification was performed with the open-source analysis platform ImageJ (cell counter plugin). Division of SNc and VTA was based on the axon bundle (medial lemniscus) separating the two areas. The number of positive cells for each animal was manually counted by a researcher blinded to experimental groups and expressed as % of control (sham-lesion).

### 2.6. Western Blotting

Mice were killed by decapitation and striata were dissected out on an ice-cold surface either freehand (with left and right hemispheres combined or kept separately) or by punches of striatal tissue (1 mm thickness, 2 mm diameter; three punches per hemisphere (divided into dorsal and ventral, Supplementary Figure S1b). The tissue was sonicated in 1% sodium dodecyl sulfate and boiled for 10 min. Equal amounts of protein (25 μg) for each sample were loaded onto 10% polyacrylamide gels and separated by electrophoresis and transferred overnight to nitrocellulose membranes (Thermo Fisher, Stockholm, Sweden). The membranes were immunoblotted with primary antibodies against actin (1:30,000, Sigma Aldrich, Stockholm, Sweden) and TH (1:2000, Millipore, Darmstadt, Germany). Detection was based on fluorescent secondary antibody binding (IR Dye 800CW and 680RD, Li-Cor, Lincoln, NE, USA) and quantified using a Li-Cor Odyssey infrared fluorescent detection system (Li-Cor, Lincoln, NE, USA). The TH protein levels were normalized for the amount of the corresponding actin detected in the sample and then expressed as a percentage of the control (sham-lesion).

### 2.7. Statistics

Statistical analyses were performed using GraphPad Prism (v. 7.00, GraphPad Software, La Jolla, CA, USA). Data are presented as mean ± SEM unless stated otherwise. Statistical significance is presented as: * *p* < 0.05, ** *p* < 0.01, *** *p* < 0.001. Text contains exact *p*-values and number of animals and/or replicates. For 2-group comparisons, one- or two-tailed Student’s *t*-tests (paired or unpaired as appropriate) were used. For 3 or more groups, a one-way ANOVA, followed by multiple comparisons with Sidak’s (multiple pairs) or Dunnett’s (vs. sham group) or its nonparametric analogue Kruskal–Wallis test, followed by Dunn’s rank difference was used. For experiments with multiple groups and longitudinal data, two-way ANOVA, followed by Holm–Sidak’s (vs. sham for each time point) test, was performed. If the data did not follow required statistical assumptions, nonparametric tests were used. The D’Agostino–Pearson Omnibus K2 normality test was used to access if data sets followed a Gaussian distribution. This test first computes the skewness and kurtosis to quantify how far the distribution is from Gaussian in terms of asymmetry and shape. It then calculates how much each value differs from the expected value within a Gaussian distribution and computes a single *p* value from the sum of these discrepancies.

Survival analyses were performed using the Kaplan–Meier method [53] followed by the log-rank Mantel–Cox test and presented as a percentage of survival, while probability of survival was calculated using logistic regression (logit) with the solver method and presented as the probability of survival. Relative changes in weight between surgery day and W1 were calculated individually (as %) and outliers were identified by a normal-quantile plot (bin 2.5%, 95% CI).

**Table 2:** Perioperative health assessment and procedures.

Type	Identification	Treatment	Reference
**General Health**	Appearance and activity pattern of the animal in home cage including interaction with environment, cage mates and nest building.	Further hands-on examination.	[54]
**Body Condition**	Visual assessment of body shape and hands-on examination by passing the fingers over sacroiliac bones.	Assess body condition score and further examine with attention to humane endpoints.	[55]
**Pain or Distress**	Mouse shows reluctance to move, failure to groom and unkempt appearance of fur coat, lack of appetite and thin body condition, loss of nest-building behavior, in some cases vocalization. Determined by mouse facial expression orbital tightening (squinting), nose bulging, cheek bulging, drawing of the ears back behind the head.	Administration of buprenorphine and consideration of euthanasia based on humane endpoints.	[56,57]
**Dehydration**	Weak appearance with recessed eyes and fuzzy fur. Skin turgor is reduced and pinched skin over the back remains tented.	Subcutaneous or intraperitoneal fluid replacement therapy with warmed Lactated Ringer’s solution, sterile saline (0.9%) or glucose (5%).	[47]
**Hypothermia**	Animals are cool at touch and have a body temperature lower than 36.5 °C.	Increase in ambient temperature of housing, cages placed in warming cabinet or on a thermal blanket, administration of warm fluids.	[58]
**Aphagia and Adipsia**	Measurement of food and water consumption. Body weight monitoring.	Easy access to palatable food supplementation, hydration complemented with glucose solution.	[59,60]
Penile Prolapse (Paraphimosis) *	Swollen, distended and reddened penis.	Lubrication and placement of soft bedding to decrease swelling, hydration support.	[49,54]
Urethral Obstruction (urethral plugs) *	Firm, cream-colored proteinaceous material observed at tip of penis.	Remove plug with lubrication and local analgesics or short anesthesia.	[61,62]

* urologic syndrome [63].

The brain heat map image presented in Figure 2d was created based on the average z-score values of sham and lesion mice for which coronal sections had been mapped (see section on Immunohistochemistry and Image Analysis). Next, for each mouse, both hemispheres were mirrored (*n* = 2 replicates/mouse) and then z-scored. Differences between groups (*n* = 5/group) were plotted as sham normalized % of TH-loss for each x,y (dorsovental, mediolateral) position within the boundaries of the striatum and heatmap-colored for presentation.

## 3. Results

### 3.1. Effect of the Bilateral Partial 6-OHDA Lesion on TH Immunoreactivity

The 6-OHDA injection in the dorsal striatum (Figure 2a) was examined in C57Bl/6 mice 3 weeks after surgery, using TH as dopaminergic marker (Figure 2b–f, Supplementary Figure S1a,b). Immunohistochemical analysis of brain sections along the rostro-caudal axis indicated that the reduction in TH-immunoreactive fibers was preferentially localized in the dorso-lateral part of the striatal formation (Figure 2b). Western blot analysis of the whole striatum, including the ventral part (nucleus accumbens) showed an overall 56.4 ± 4.7% decrease in TH levels (Figure 2c, left) (sham = 100 ± 4.9 (*n* = 8 mice) and lesion = 43.56 ± 12.8 (*n* = 14 mice); unpaired one-tailed *t*-test, *p* < 0.0001). The effect of the lesion was similar in the two hemispheres, with nearly identical decreases in TH-immunoreactivity in left (60.5 ± 5.1%) and right (57.3 ± 5.3%) striata, in comparison with the respective ipsilateral control. (Figure 2c, center) (sham *n* = 8, lesion *n* = 12; ratio paired *t*-test for lesion left vs. right hemisphere, two tailed, *p* = 0.5267, t = 0.6538, df = 11).

We then assessed TH levels in the dorsal and ventral striatum. We observed a reduction of 79.5 ± 3.8% in dorsal and 12.3 ± 4.0% in the ventral striatum of lesion mice as compared to sham (Figure 2c, right) (sham = 20 lesion = 24, each region evaluated separately; One-way ANOVA, F_(2, 65)_ = 239.7, *p* < 0.0001. Sidak’s; sham^dorsal^ vs. lesion^dorsal^ *p* < 0.0001, sham^ventral^ vs. lesion^ventral^, *p* = 0.0091, lesion dorsal vs. ventral, *p* < 0.001). These findings were corroborated by mapping the extent of the 6-OHDA lesion at the level of the injection site (Figure 2d). We found that the lesion reached maximal TH-fiber depletion in the dorso-lateral striatum (yellow) with only a negligible effect observed in more medial striatal areas (i.e., dorso-medial, dark blue). Ventrally, the lesion reached the border of the nucleus accumbens but remained mostly restricted to the shell region. Taken together, these data indicate that the volume infused had limited diffusion and produced a restricted area-of-effect.

Finally, we examined the impact of the partial lesion on cell loss within the SNc and the VTA, which are the two major dopaminergic afferents to the striatal formation. Immunohistochemical analysis of the rostro-caudal extent of these regions showed a 59.5 ± 6.8% loss of dopaminergic neurons within SNc, which project mostly to the dorso-lateral striatum (in %, sham = 100 ± 4.7 and lesion 40.49 ± 4.9 (*n* = 4 mice per group/8 sections per mice), unpaired one-tailed *t*-test, *p* < 0.0001, t = 8.757, df = 6). In contrast, the VTA, which projects preferentially to the dorso-medial and ventral striatum, showed only a 10.8 ± 5.3% decrease in TH-positive cell number (sham = 100 ± 1.3 and lesion 89.12 ± 5.2 (*n* = 4 mice/8 sections each), unpaired one-tailed Student’s *t*-test, *p* = 0.0434, t = 2.041, df = 6). Overall, these results indicate that the neurotoxic effect of 6-OHDA is for the most part limited to the dorso-lateral striatum and its dopaminergic afferents from the SNc (Figure 2e,f).

### 3.2. Effect of SC and EC Protocols on Survival

We next compared the effect of the SC and EC protocols on 6-OHDA-induced mortality. A total of 1254 mice, utilized over 7 years, were included in this study. Mice were injected in the dorsal striatum with 6-OHDA (lesion mice) or an equivalent volume of vehicle (control, sham-lesion mice). The SC protocol consisted of a combination of interventions commonly applied to animals subjected to stereotaxic surgery (Figure 1, top), in line with ethical requirements from the Directive 2010/63/EU on protection of animals used for scientific purposes. The EC protocol was designed based upon those same directives, with several additional pre- and post-surgical interventions (Figure 1, bottom). The applied changes were based on published reports and personal communication with researchers who also reported mortality when performing 6-OHDA lesion [64,65,66,67].

**Figure 3:**
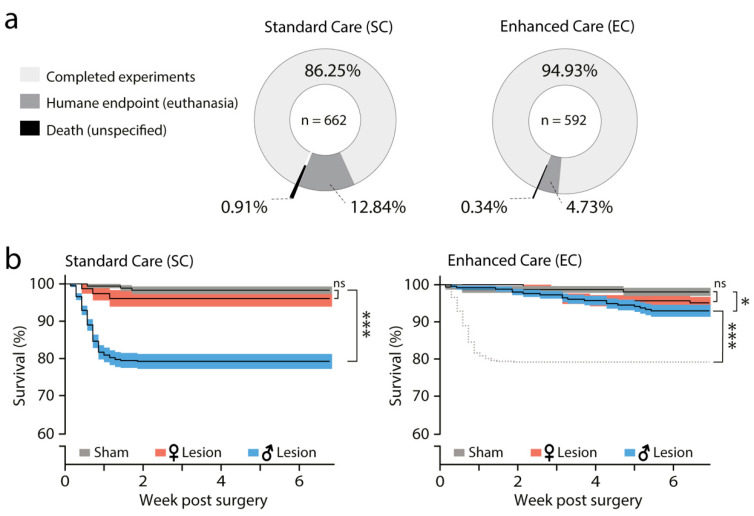
Enhanced perioperative care improves survival following bilateral injection of 6-OHDA. (**a**) Pie charts showing the post-surgical outcomes of SC and EC—the percentage of animals that survived and were then used for experimentation (light grey) or were lost during post-surgical recovery (dark grey/black). (**b**) Left, SC survival curves (%; Kaplan–Meier method lines and asymmetrical SEM, shadow zones) of sham (grey, *n* = 176), female^lesion^ (red, *n* = 77) and male^lesion^ (blue, *n* = 409) mice. Mantel-Cox test (*p* < 0.0001) followed by log-rank analyses of pairs showed higher mortality risk for male^lesion^ group (sham vs. male^lesion^, *p* < 0.0001, sham vs. female^lesion^, *p* = 0.2871). Right, EC survival curves of sham (grey, *n* =155), lesion female (red, *n* = 181) and male^lesion^ (blue, *n* = 256) mice. Dashed line represents male^lesion^ from SC protocol (SC-male^lesion^). Mantel-Cox test (*p* = 0.0749) followed by log-rank analyses of pairs showed reduction in mortality risk for the EC-male^lesion^ group (EC-sham vs. male^lesion^, *p* = 0.0243, EC-male^lesion^ vs. SC-male^lesion^, *p* < 0.0001, sham vs. female^lesion^, *p* = 0.1487). * *p* < 0.05, *** *p* < 0.001.

Each protocol included nearly half of all examined mice [52.8% in SC (*n* = 662) and 47.2% EC (*n* = 592)]. We used both male (*n* = 805) and female (*n* = 449) C57Bl/6 mice which were 2.5 to 4 months old at the time of surgery. In the SC group, we found a mortality of 13.75% (91/662), which was reduced to 5.07% (30/592) in the EC group (Figure 3a). Most losses were associated with humane end point decisions, 12.84 and 4.73% in the SC and EC group, respectively.

Lesion and sham mice were then divided by gender and their survival curves analyzed using the Kaplan–Meier method [53]. We observed rare death events in control mice (6/331 both protocols joined), which were not associated with gender or care-protocol. Therefore, we combined all control mice (Figure 3b, grey) from each protocol (SC mice = 176 and EC mice = 155) and compared sham survival curves with those of 6-OHDA lesion mice by gender (referred to as male^lesion^, female^lesion^).

In SC, comparison of sham-lesion with male^lesion^ (blue) and female^lesion^ (red) mice showed a significant difference in survival between sham (98.3%) and male^lesion^ (79.2%) (Matel-Cox test, *p* < 0.0001, Chi_square_ = 44.34, df = 2), but not female^lesion^ (96.1%) mice. Paired analysis revealed that male^lesion^ mice are at a much higher risk of loss than female^lesion^ mice (Logrank analyses, sham vs. male^lesion^, *p* < 0.0001, Chi_square_ = 34.05, df = 1 and sham vs. female^lesion^, *p* = 0.2871, Chi_square_ = 1.133, df = 1) (Figure 3b, left).

In contrast, when mice were subjected to EC, male^lesion^ mice showed a significant drop in mortality (from 79.2% in SC to 92.3% in EC) and hazard ratio (Mantel-Henszel, SC/EC = 2.9, CI = 1.9 to 4.4). Paired analyses of SC-male^lesion^ vs. EC-male^lesion^ showed that the survival curve was improved when animals were treated according to the EC protocol (Logrank, *p* < 0.0001, Chi square = 25.93, df = 1). Despite this significant improvement, comparison of the survival of EC-sham (98.1%) mice with that of EC-male^lesion^ (92.3%) and EC-female^lesion^ (95.1%) mice indicated slightly higher mortality risk for males (Logrank analyses, sham vs. EC-male^lesion^, *p* = 0.0243, Chi square = 5.076, df = 1 and sham vs. EC-female^lesion^, *p* = 0.1487, Chi square =2.085, df = 1) (Figure 3b, right).

In conclusion, these results show that upon 6-OHDA infusion, male^lesion^ mice have a remarkably higher risk of post-surgical death then female^lesion^, and that EC nearly abolishes mortality in 6-OHDA male^lesion^ mice.

### 3.3. Survival of 6-OHDA Male^lesion^ Mice Depends on Age and Can Be Predicted by Weight

**Figure 4:**
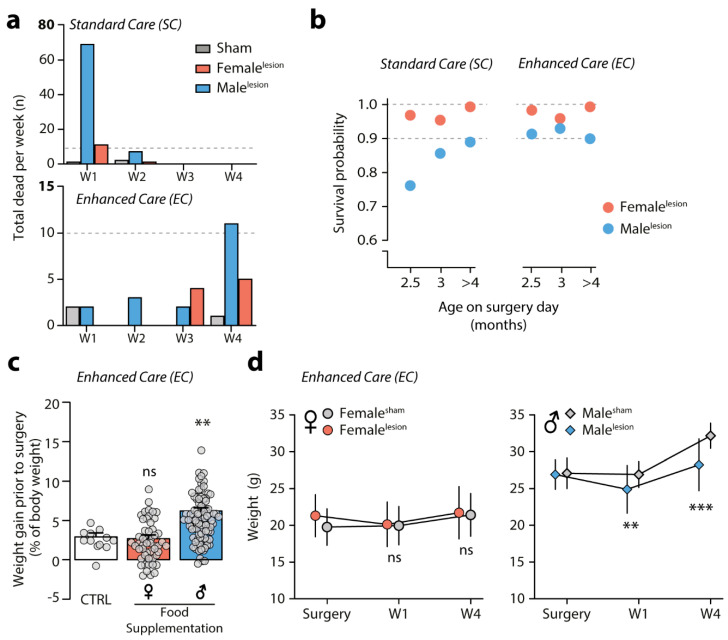
6-OHDA lesion-induced weight variability affects survival. (**a**) Bar graphs showing the number of animals lost in the weeks (W) following striatal lesion in SC and EC groups. SC loss 91/662 and EC loss 30/592. (**b**) Survival probability for female^lesion^ (red, *n* = 122 and 286) and male^lesion^ (blue, *n* = 448 and 310) mice receiving SC and EC, calculated by Logistic Regression Logit, showing difference in predictor factor ‘age’ before and after the implementation of EC protocol. (**c**) On the week prior to surgery, EC protocol promotes weight gain in males (blue, *n* = 91) but not females (red, *n* = 51) as compared to control (normal chow; CTRL, *n* = 12). Data are presented as mean ± SEM (Kruskal–Wallis test, *p* < 0.0001, statistic = 32.27. Dunn’s rank difference, CTRL vs. EC-female *p* > 0.9999, CTRL vs. EC-male *p* = 0.0066). (**d**) Line graphs (mean ± SD) showing weight (g) on surgery day, W1 and W4 post-surgery, in mice treated with EC which survived (female^sham^ *n* = 73 ± 6; female^lesion^ *n* = 120 ± 24; male^sham^ *n* = 25 ± 4; male^lesion^ *n* = 139 ± 20). Females (left) and males (right). Females show comparable weight throughout the whole period (two-way ANOVA, interaction F_(2, 576)_ = 3.175, *p* = 0.0425, Holm–Sidak’s surgery day *p* = 0.001, W1 *p* = 8148, W4 *p* = 8148), whereas male^lesion^ mice lose weight during the post-surgery recovery period (two-way ANOVA, interaction F_(2, 486)_ = 8.884, *p* = 0.0002, Holm–Sidak’s surgery day *p* = 0.7425, W1 *p* = 0.0015, W4 *p* < 0.0001). ** *p* < 0.01, *** *p* < 0.001.

The survival curves (presented in Figure 3b) indicate that most losses occur within the first week post-surgery. In Figure 4a, we plotted the number of deaths/group/week for SC mice, showing that 81 out of a total of 91 deaths occurred during W1. In contrast, we found that for EC mice, the number of deaths is much lower and tends to occur on W4 after surgery (17/30 deaths). Although the total number of deaths greatly differs between protocols, these data suggest that EC promotes survival mainly by reducing mortality during the most critical post-surgery period (i.e., W1). Knowing that SC-male^lesion^ are at highest risk during W1 raises the question of why males are susceptible to death upon 6-OHDA injection even though surgical procedure and lesion severity between genders is identical (TH striatal, Western blot analysis between genders: two-way ANOVA, gender F_(1,50)_ = 0.6863, *p* = 0.4114, Holm–Sidak’s female^lesion^ vs. male^lesion^ *p* = 0.3683, *n*= 16 females/group; *n* = 8 male^sham^, 14 male^lesion^, data not shown).

Hence, we decided to investigate other variables that could influence survival. We ran a regression analysis using a logistic model (Logit, SC *n* = 570/662 and EC *n* = 592/592 mice included) to assess the effect of gender, age at surgery (in months) and surgeon experience (measured as number of batches performed, *n* = 6 surgeons) on the probability of death. We found that provided the surgeon had received initial technical training in stereotaxic surgery, surgeon experience had no effect on survival regardless of care-protocol (predictor *p*-value, SC_surg_ = 0.1 and EC_surg_ = 0.741). The main variable influencing survival was, as expected, 6-OHDA-lesion vs. sham-lesion, but this was only the case for mice receiving SC (predictor *p*-value for SC_lesion_ = 1.094 × 10^−6^ and for EC_lesion_ = 0.515). Additionally, in SC both gender and age were factors that influenced survival (SC_gender_ = 0.0006, SC_age_ = 0.0085). In contrast, with EC, neither gender nor age were significant factors (EC_gender_ = 0.343, EC_age_ = 0.985). By comparing survival probability by gender and age, we identified young SC-male^lesion^ (2–3 months old at the time of surgery) as the subgroup of males with highest risk of post-surgical death, whereas for EC-male^lesion^, this age subgroup was not at increased risk (Figure 4b).

Thus far, the variables included in our model could not identify which subgroup of mice receiving EC was at higher risk. The EC protocol included daily weight monitoring. Therefore, we added this parameter to our logistic model. We found that weight (g) on surgical day was the only factor that could predict survival within this protocol (EC_weigth_ < 0.0001).

Since the EC protocol included access to food supplementation during the week before surgery, all groups of mice were expected to gain weight. Therefore, we analyzed weight gain on the week prior to surgery for experimental mice and a control group fed only with standard chow. We found that females gained weight to the same extent as standard chow control mice (CTRL, *n* = 12; gain 2.9 ± 0.49%. EC_female_, *n* = 51 gain 2.7 ± 0.45%), whereas male mice gained on average three times more body mass (EC_male_, *n* = 91, 6.2 ± 0.38%) within that same pre-operative week (Figure 4c) (Kruskal–Wallis test, *p* < 0.0001, statistic = 32.27. Dunn’s rank difference, CTRL vs. EC_female_ *p* > 0.9999, CTRL vs. EC_male_ *p* = 0.0066). These results were in line with the literature reporting that female C57BL/6 mice are resistant to diet-induced weight gain as compared to male mice [68].

Next, we assessed weight during the post-operative weeks in EC for sham and lesion mice all of which were fed with supplementary food. Here, we compared the weight on surgery day with that at the end of W1 and W4 (Figure 4d) (on average, per group: female^sham^ *n* = 73 ± 6; female^lesion^ *n* = 120 ± 24; male^sham^ *n* = 25 ± 4; male^lesion^ *n* = 139 ± 20). We observed that both females and males underwent surgery with a similar weight relative to their gender (mean difference ± SEM; female = 1.5 ± 0.39 g, male = −0.18 ± 0.41 g). In line with the fact that pre-surgical weight was not used to select which mice underwent 6-OHDA lesion. At the end of W1, male^lesion^ mice were lighter than their counterparts and by W4 the difference between male sham and lesion groups was 3.9 ± 0.68 g (two-way ANOVA, interaction F_(2, 486)_ = 8.884, *p* = 0.0002, Holm–Sidak’s surgery day *p* = 0.7425, W1 *p* = 0.0015, W4 *p* < 0.0001). In contrast, female^lesion^ showed comparable weight throughout the weeks, with the mean difference between groups below 1.5 ± 0.42 g (Two-way ANOVA, interaction F_(2, 576)_ = 3.175, *p* = 0.0425, Holm–Sidak’s surgery day *p* = 0.001, W1 *p* = 8148, W4 *p* = 8148).

Knowing that post-operative weight can vary and that weight on surgery day is a factor that influences survival in EC, raises the possibility that individual weight change can be used as a tool to identify animals at risk of reaching humane endpoint by W4. Thus, we calculated individual weight variation (% of g change) between surgery day and W1 for all animals in EC. We used normal-quantile plots to identify outliers on W1 and determine if those animals would end up reaching humane endpoint by W4. We identified a total of 50/592 outliers, all of which were lesion mice with weight loss ranging from 15.13 to 23.67% in W1 (overall mean 3.91% with CI 3.16 to 4.66%). Amongst those were 16/17 mice that reached humane endpoint by W4. Therefore, by identifying male^lesion^ that lose more than 15% of their surgery weight on W1, one can find the subgroup of males within which nearly all male^lesion^ deaths will occur.

Finally, we examined the possibility that cage group number could negatively affect the recovery process of male^lesion^ mice by reducing or precluding access to supplementary food. We found no effect of group number on survival rate between males nor females (Supplementary Figure S1c) (% of deaths; two-way ANOVA, gender F_(1,2)_ = 0.6457, *p* = 0.5060, cage size F_(2,2)_ = 7.791, *p* = 0.1138). Importantly, the EC protocol includes regular health surveillance, which allows the researcher to identify mice in need of special care. Out of the 256 male^lesion^ mice on our EC protocol, 75% (192/256) had an uncomplicated recovery, whereas 25% (64/256) had at least one post-surgical complication. In these animals, the occurrence of urethral plugs was by far the most frequent, representing 68.5% of all complication cases (44/256 mice, 44/64 complication cases). This shows the importance of health surveillance for male^lesion^ mice, to reduce the negative impact of obstructive uropathy (Supplementary Figure S1d–f and Table 2). These results indicate that the first week after 6-OHDA administration is critical for the recovery of male^lesion^ mice and that the EC protocol significantly reduces the loss male^lesion^ mice operated at 2.5–3 months of age, which are the experimental group at higher risk of mortality. In the same high-risk group, weight gain as a result of pre-operative food supplementation appears to be a critical factor for effective post-surgical recovery.

## 4. Discussion

In this study, we describe a protocol for the generation of a toxin-based mouse model of bilateral, early-stage parkinsonism, particularly indicated for the study of non-motor symptoms. In comparison to other toxins, 6-OHDA produces a robust and stable lesion, lasting for several weeks [30,37,48,69], without the reversing and compensatory responses reported, for instance, after administration of 1-methyl-4-phenyl-1,2,3,6-tetrahydropyridine (MPTP) [70,71,72].

In contrast to MPTP, which is most often administered systemically, 6-OHDA does not cross the blood–brain barrier and must be surgically microinjected in selected brain regions. The combination of this invasive procedure with the concurrent neurodegenerative processes is at the basis of the high mortality observed in the 6-OHDA model [64]. To reduce this problem, mice with a unilateral 6-OHDA lesion in the MFB have been treated with post-surgical interventions, including supplemental nutrition, rehydration, and external temperature control. Various combinations of these procedures decreased mortality to 20–10% [65,66,67] and in some cases even eliminated it [73,74]. In the present model, a similar standard preventive care protocol resulted in a 79.2% survival rate in males and 96.1% in females. This difference is likely linked to the persistent blocking of the urethra and penis prolapse in males observed during the weeks following surgery, which may lead to chronic obstructive uropathy. The mechanism by which intracerebral injection of 6-OHDA results in this peripheral complication remains to be elucidated. We concluded that the more severe invasive character and broader effect of the bilateral 6-OHDA injection required a more extensive EC protocol. Indeed, pre-operative food supplementation combined with more frequent daily checks to prevent urologic complications increased the male survival rate to 92.3%.

The dramatic difference in the response of male vs. female mice to the SC protocol, with females displaying a survival rate similar to control mice, suggests that the use of this gender is preferable in combination with a simpler care protocol. Notably, gender has been shown to correlate with the propensity to develop specific non-motor symptoms and with their severity. For instance, affective disorders, constipation and pain have been more frequently diagnosed in women, whereas men show a higher susceptibility to developing cognitive disorders and sleep and sexual disturbances [75,76,77,78]. These findings should be kept in mind when examining the phenotype produced by the bilateral lesion in mice of different gender.

PD is still commonly diagnosed based on the manifestation of unilateral motor symptoms. However, positron emission analysis of early-stage patients showed a bilateral reduction in striatal dopaminergic markers, which may precede the cardinal motor symptoms and concur to the manifestation of prodromal comorbidities [79]. Keeping this in mind, one advantage of the partial striatal lesion is its compatibility with bilateral toxin administration, which is more difficult to accomplish with 6-OHDA injection in the MFB.

The bilateral lesion described in this study does not affect locomotion in the open field [21] and produces only minor motor deficits, limited to vertical activity [21] and hindlimb gait coordination [40]. Importantly, mice with this lesion display intact motor activity during cognitive and affective tests [21,41], including those necessary to assess non-motor symptoms associated with PD [21,39,40,41]. The relatively small changes in motor function observed in this lesion are accompanied by reductions in dopamine neurons (≈60%) and striatal dopamine innervation (70–75%) similar to those occurring in PD patients at the onset of motor symptoms [80], further validating the early-stage character of this model. The impairment of the nigrostriatal system produced by striatal injection of 6-OHDA is accompanied by a more limited damage in the VTA and nucleus accumbens, which is in line with the relative sparing of these structures observed in PD patients [81,82]. It should be kept in mind, however, that despite their more moderate character, the neuronal loss in the VTA and the associated impairment of the mesocorticolimbic dopamine system may contribute to cognitive and affective comorbidities [83,84].

Based on these observations, and on its mild motor phenotype, the PD model described in this study represents an attractive tool for the behavioral and mechanistic investigation of non-motor symptoms. Indeed, several studies have shown how the partial bilateral lesion of the dorsal striatum is associated with the manifestation of a wide range of conditions that recapitulate comorbidities observed in PD patients.

Initial work in CD1 mice with a bilateral lesion of the dorsal striatum showed impaired memory, detected using the object recognition test. This effect appeared to be limited to spatial, but not object recognition [38]. In contrast to this latter finding, other studies reported impaired novel object recognition in C57Bl/6 mice with a similar lesion [21,39,41]. This discrepancy may be explained by the different mouse strains utilized in these studies, or by differences between the behavioral protocols, and should be considered in future investigations on this specific subject.

The present model of PD showed increased immobility, indicative of depressive-like behavior, when examined with the forced swim and tail suspension tests [21,41]. This abnormality was prevented by sub-chronic (4 days) administration of the dopamine receptor agonist, pramipexole, but was refractory to L-DOPA [40], in line with observations in patients subjected to similar dopamine replacement therapies [85]. Increased immobility in the forced swim test was also observed in mice with a nearly complete bilateral lesion produced by 6-OHDA injection in the substantia nigra. In this case, depressed behavior was counteracted by long-term (20 weeks) administration of both pramipexole and L-DOPA [86]. Anxiety has also been observed in the partial bilateral model of PD, using standard behavioral paradigms such as the elevated plus maze and the light-dark box tests [39,40,41].

It should be noted that earlier work using a bilateral mouse model of PD characterized by a 40% depletion of striatal dopamine did not report changes in spatial memory. Moreover, this model reduced, rather than promoted, depressive- and anxiety-like behavior [37]. The results of these studies indicate the importance of controlling the severity of the 6-OHDA lesion, which should result in a depletion of striatal dopamine of at least 60% [21,38].

In addition to cognitive and affective abnormalities, the model described in this study was found to reproduce olfactory deficit [40], which is a particularly frequent prodromal symptom in PD patients [4,5]. Impaired odor discrimination was detected, both between different non-social odors and different social odors [40], using the habituation-dishabituation protocol [87].

Abnormal rest–activity circadian rhythm, accompanied by sleep disturbances, is also frequently observed in early PD [88]. Interestingly, mice with a partial bilateral 6-OHDA lesion maintained in darkness displayed a severe fragmentation of the rest–activity cycle, indicative of disrupted endogenous circadian rhythm [39]. In addition, these animals showed reduced locomotion during the active phase of the circadian cycle [39], suggestive of excessive daytime sleepiness, which is frequently observed in PD patients.

## 5. Conclusions

Partial bilateral lesion of the dorsal striatum with 6-OHDA has been previously employed to reproduce multiple non-motor symptoms commonly associated with PD. The use of this model is complicated by high mortality, particularly among males, which can be nearly eliminated by applying a number of pre- and post-surgical interventions. Reduction in animal loss will facilitate the use of the model to study non-motor comorbidities, which represent an urgent clinical problem in the management of PD.

## Figures and Tables

**Table 1:** Motor and non-motor behavioral profile of the bilateral partial lesion model described in this article.

Domain		Test	Phenotype	Pharmacological Intervention	References
**Motor**	**Spontaneous locomotion**	Open field	No effect	-	[40,41]
		Novel home cage	No effect	-	[21]
	**Vertical activity**	Cylinder	Reduced rearing	-	[21]
	**Gait pattern**	Treadmill (ventral plane videography)	Impaired hindlimb gait dynamics	-	[40]
**Affective**	**Depression**	Porsolt forced swim	Increased immobility	L-DOPA = Pramipexole + Reboxetine + Rapamycin +	[40,41]
		Tail suspension	Increased immobility	-	[40]
	**Anxiety**	Open field center zone	Center avoidance (thigmotaxis)	Rapamycin +	[40,41]
		Elevated plus maze	Reduced exploration of open arms	L-DOPA = Pramipexole + Reboxetine + Thioperamide = Rapamycin +	[39,40,41]
		Light-dark box	Increased latency to enter the bright chamber	Thioperamide =	[39]
**Cognitive**	**Novelty detection**	Novel object recognition	Deficit in long-term recognition memory	L-DOPA + Pramipexole = Thioperamide + Rapamycin +	[21,39,41]
**Circadian**	**Circadian activity**	Circadian activity rhythm in social environment	Reduced activity during the active period of the 24 h cycle	Thioperamide +	[39]
		Endogenous activity cycle in social environment during constant darkness	Disruption of the endogenous circadian rhythm (activity pattern fragmentation)	-	[39]
**Olfactory**	**Olfactory discrimination**	Olfactory habituation/dishabituation	Deficit of olfactory discrimination	-	[40]

+ indicate recovery; = lack of effect; - not investigated.

## Data Availability

Raw data is available upon reasonable request.

## Supplementary Materials

The following are available online at https://www.mdpi.com/article/10.3390/biomedicines9060598/s1, Figure S1: Support data regarding 6-OHDA lesion and post-surgical care. Supplementary Data: Step-by-Step description of surgical procedure.
