# Supplementary material for: A Guide to the Generation of a 6-Hydroxydopamine Mouse Model of Parkinson’s Disease for the Study of Non-Motor Symptoms
Source: Biomedicines. 2021 May 25;9(6):598. doi: 10.3390/biomedicines9060598 (PMC8227396; doi:10.3390/biomedicines9060598)
Supplement: Supplementary file 1 [file biomedicines-09-00598-s001.zip › biomedicines-1187184-supplementary.pdf]

Supplementary Figure S1

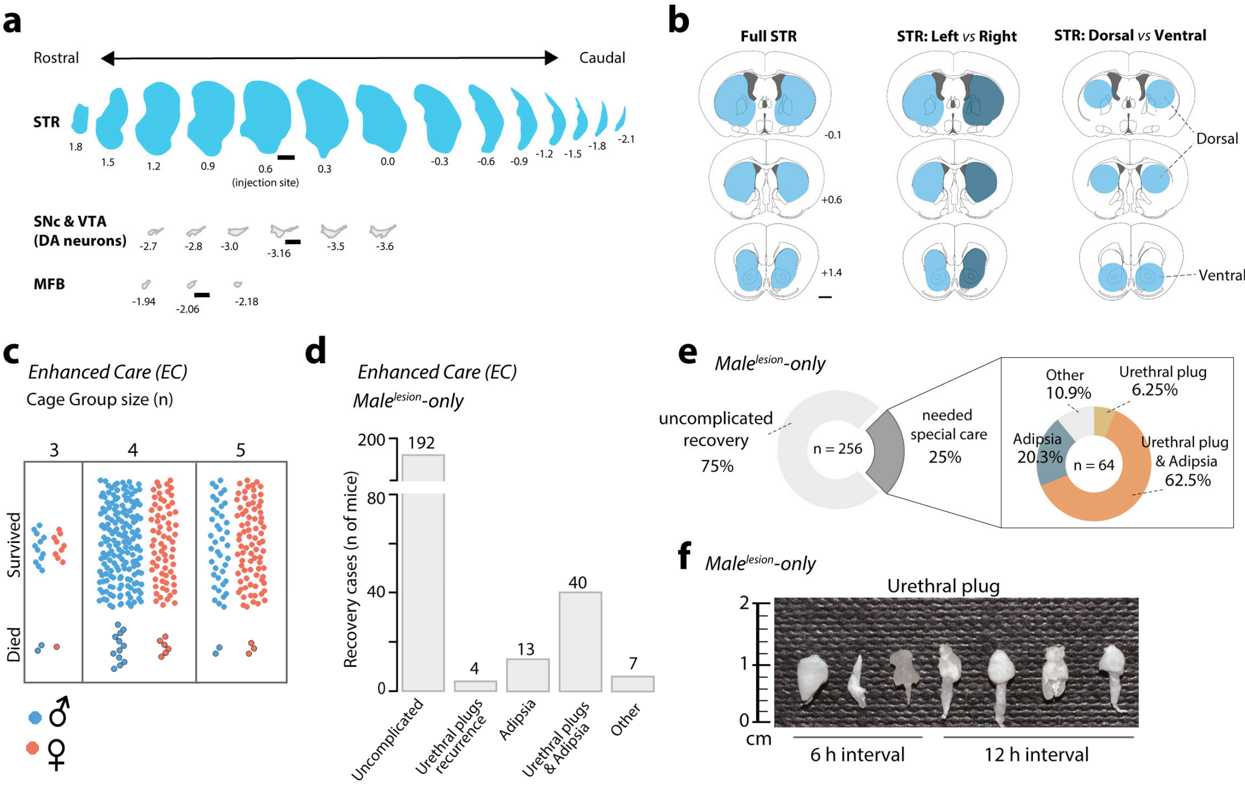

Supplementary Figure S1. Support data regarding 6-OHDA lesion and post-surgical care. (a) Common targeted sites for 6-OHDA infusion. Schematic showing the relative size of the 3 possible targets. Note the much larger size of the striatum (STR), in comparison to SNc, VTA and medial forebrain bundle (MFB), making this structure a preferential target for models of PD based on partial 6-OHDA lesion (scale 1mm). **(b)** *Upper panel*, schematic of the dissections used to quantify TH in this study: free-hand whole striatal sampling combined (left) or separated by hemisphere (center), and microdissection of dorsal and ventral striatum with punching using dissection needles (right). **(c)** Cage group size and mortality in a subset of 403 mice (26 losses in total). No association between gender, group size and percent of loss was found. Male loss: cage of 3=2/14 (14%), cage of 4=12/180 (6.7%), cage of 5=2/42 (4.8%). Female loss: cage of 3=1/10 (10%), cage of 4=6/74 (8.1%), cage of 5=3/80 (3.6%) **(d)** Health surveillance and annotated cases during post-surgical recovery of male^{lesion} mice in enhanced care (EC) protocol. **(e)** Presentation of data in **d** as percentage of cases. Amongst a subset of 256 mice male^{lesion} mice, 75% of all mice had an uncomplicated recovery (192/256). Whereas 25% had at least one complication incidence. Amongst possible complications, urethral plugs are present 68.5% of all complication cases [re-occurrence, 6.25% (n=4) + 62.5% (n=40)]. Note how most urethral plugs are concomitant to adipsia (*i.e.* urologic syndrome). **(f)** Images show examples of urethral plugs (*i.e.* obstructive uropathy), which can develop fast over the course of hours, requiring daily checks. **d-f**; for definitions regarding health assessment see Table 2**.** 6-OHDA: 6-hydroxydopamine, TH: tyrosine hydroxylase, SNc: substantia nigra compacta, VTA: ventral tegmental area.

Supplementary Data

Surgical Procedure Step-by-Step

Surgical environment and aseptic technique

The surgical workspace should be kept clean, organized and out of laboratory traffic flow. Predefined functional areas for preparation of the animal, surgical procedure and recovery will facilitate work and minimize potential contamination of the sterile field. It is helpful for the surgeon to have an assistant to help with anesthesia, preparing animals, and monitoring recovery.

Surgery is to be performed under an appropriate airflow, such as a vertical laminar hood or a downdraft table, allowing disposal of extraneous anesthetic gas and contaminant particles.

All areas must be cleaned and disinfected with 70% ethanol and surgical instruments must be sterilized prior to the procedure. When performing batch surgery, it is advisable to have more than one set of sterile instruments or sterilize the instruments between animals using a hot glass bead sterilizer. Surgeons should wear a surgical gown and cap, a facemask and sterile surgical gloves.

Preparation of the animal should occur in a location different than that used for performing the surgery. Cage bedding should be covered with a paper towel to avoid the airways from being obstructed while the mouse regains consciousness. Position half of the cage on a heating pad to avoid hypothermia (anesthetics can depress thermoregulatory activity).

Preparation of surgical equipment

The stereotaxic frame (Stoelting Europe, Dublin, Ireland) should be placed in the center of the airflow hood. Materials such as: drill (equipped with drill tip HM71 007, Foredom, Agnthos, Lidingö, Sweden,), electronic heating pad, light source, sterile cotton pads or cotton tips, an infusion pump (KD Scientific, Agnthos, Lidingö, Sweden), a Hamilton syringe (5 to 10 μL), a surgical microscope (Zeiss Opmi 1), and all products/instruments necessary for surgery must be within reach and considered as part of the sterile field.

1. The instruments should be placed on a sterile gauze in order of use: surgical scissors, DeBakey straight atraumatic forceps (tongue manipulation), curved splinter forceps (tissue manipulation, skull prodding), tissue forceps (wound closure), watchmaker forceps and scalpel blade.

2. Fix the micro injector needle (36-gauge, Coopers Needle Works Ltd, Birmingham, England) to the stereotaxic probe holder and connect to the Hamilton syringe via TYGON plastic tubing (Formulation R-3603, Ø 0.19 mm, Cole Palmer). The Hamilton syringe, plastic tubing and injector must be filled with sterile mineral oil (Sigma-Aldrich, Stockholm, Sweden). Alternatively, the use of glass capillaries (Wiretrol I, Broomall, PA) with an automated stereotaxic injector (Quintessential, Stoelting Europe, Dublin, Ireland) is recommended.

3. Turn on the drill and adjust the speed. Make sure the drill bit rotates at low speed to avoid thermal injury to the bone tissue.

4. Turn on heating pad and adjust to 37 °C.

5. Make sure the stereotaxic frame is ready to receive the anaesthetized animal. Fix only one ear bar approximately 3 mm from the midline of the apparatus. The other bar is kept loose on the side until placed.

6. The Hamilton syringe is placed on the infusion pump holder and the pump is set to infuse 1 μL at a rate of 0.2 μL/min.

Preparation of the solutions

7. Dissolve 6-OHDA in sterile saline (0.9%) with ascorbic acid (0.02%) (Sigma-Aldrich, Stockholm, Sweden) at 4 μg/μL (free base) final concentration. The 6-OHDA solution is light and heat sensitive and should therefore be kept on ice covered in aluminum foil. Aliquots must be prepared fresh prior to surgery and should be used within 4 h. Oxidation of 6-OHDA, which results in a color change from transparent to pink/brown, makes it no longer neurotoxic, requiring disposal and preparation of a new solution.

8. Apart from the above-mentioned material, the procedure requires: ethanol 70%, Oftagel (Santen, Apoteket, Stockholm, Sweden), Xylocain (5% Lidocain cream, Aspen, Apoteket, Stockholm, Sweden), Jodopax desinfectant (1:4 in distilled water, Apoteket, Stockholm, Sweden**),** Temgesic (0.3 mg/mL in sterile saline (0.9%) to be injected at 0.1 mg/kg, Apoteket, Stockholm, Sweden), 5 % sterile glucose solution, insulin syringes (Becton Dickinson, VWR, Spånga, Sweden) and Vetbond tissue adhesive (3M Medical, VWR, Spånga, Sweden).

Surgery

9. Set the oxygen flow on the isoflurane vaporizer to 300 mg/mmHg with 4% isoflurane delivery at 4 L/min (gas flow rate) for induction of anesthesia. Make sure the levels of isoflurane are sufficient to last throughout the entire procedure and that the necessary connectors to the induction chamber and the mouse nose mask are unobstructed.

10. Transfer the mouse to the electronic balance and record the pre-operative weight.

11. Transfer the mouse to the induction chamber. Turn on the vaporizer and observe the mouse. Anesthetic depth needs to be monitored throughout the whole procedure and can be evaluated by respiration rate, reflex response, and color of the mucous membranes. Observe the mouse in response to stimuli, as muscle movements and vocalization can indicate semi-consciousness or pain. In a state of surgical anesthesia, muscles are relaxed, and the animal is fully unconscious. The respiration rate should be slightly decreased from normal rate and the chest is moving in a slower and regular rhythm. If the rate increases during the surgical procedure, the animal is not sufficiently anesthetized, whereas gasping or irregularly breathing are a result of a too deeply anesthetized animal. Withdrawal and palpebral reflexes must be absent, while the corneal reflex might remain blunted. Pedal reflex can be assessed by pinching the web of the skin between the toes in different spots with a flexion of the limb indicating a insufficient anesthetic state. Pinching of the ear should not lead to head or whisker movements and touching of the medial canthus of the eye should not lead to blinking (palpebral reflex). Animals that are carefully handled will be anaesthetized with no undue stress. Note that the induction chamber size can affect the duration of this step.

12. When the mouse has reached a surgical anesthetic depth, remove it from the induction chamber. Holding the mouse by the scruff of the neck, use one prong from the DeBakey forceps to gently push the tongue out of one side of the mouth to free airways.

13. Place the upper teeth in the palate bar and place the anesthesia mask over the snout of the animal. Carefully find the correct position of the pre-fixed ear bar in the external auditory meatus. Afterwards, insert the second ear bar into the opposite auditory meatus, keeping it horizontal. Apply gentle pressure until the skull is fixed in a stable position. The head should then be resistant to movement in all three body planes (dorso-ventral, medio-lateral and antero-posterior).

14. Inject 10 mL/kg of Temgesic (0.1 mg/kg) subcutaneous. Apply ophthalmic ointment to both eyes to prevent corneal desiccation and repeat whenever necessary during the procedure. Check the anesthetic state and adjust isoflurane flow as needed (maintenance during surgery between 1-2%).

15. Shaving of fur should preferably be done in a separate location outside the sterile field. If not possible, fur removal within the sterile field can be performed at this stage. Use a pair of surgical scissors and collect the fur in an ethanol-wet paper towel.

16. Pass two cotton tips dipped in ethanol 70% on the surface to disinfect and capture any remaining fur. Use a third cotton tip to apply Jodopax on the same surface and wait 1 min for it to air dry.

17. Apply the local anesthetic Xylocaine to the surface of the skin. Wait approximately 5 min and ensure that the animal is deeply anesthetized and all reflexes (pedal withdrawal reflex in the forelimbs and hind limbs, the tail pinch reflex, and the eyelid reflex) are absent.

18. Incise the scalp from between the ears (posterior) to between the eyes just posterior to the margin of the occipital bone (approx. 1 cm). The periosteum should not be cut through and the skull should not be scratched. Scrape the periosteum sideways with the splinter forceps or the back of the scalpel, this precaution reduces inflammation and facilitates tissue healing (blunt dissection).

19. With the help of cotton tips and the surgical microscope, expose both lambda and bregma.

20. Zero the injector on the antero-posterior (AP), medial-lateral (ML) and dorsal-ventral (DV) coordinates of bregma. Proceed by tagging the lambda position. ML and DV measures should coincide for both bregma and lambda. If the skull is not levelled, raise/lower the palate bar and repeat the measurements. In case there are differences in ML coordinates, check the ear bar position.

21. Move the injector to the coordinates: AP + 0.6; ML + 2.2. Touch the surface of the skull with the injector needle to leave a drop of mineral oil that can act as a position marker.

22. Drill a hole (approx. 0.7 mm diameter) in the skull at the site of injection. The drill bit should not penetrate the meningeal membranes or blood vessels.

23. Confirm the initial positions of bregma and lambda after drilling.

24. Repeat steps 21 - 23 on the contralateral side adjusted for ML - 2.2.

25. Empty the Hamilton syringe of its mineral oil.

26. Break the dura on both sides with a sterile needle.

27. Dim the lights in the working area to prevent oxidation of the 6-OHDA solution. Draw up 10 μL (slowly, approx. 1 μL/s) of 6-OHDA with the injector needle.

28. Check that the injector needle is not blocked by releasing a drop of 6-OHDA.

29. Insert the injector needle slowly (0.1 to 0.3 mm/s) according to the target coordinates (AP + 0.6; ML ± 2.2 DV −3.2). Wait 1 min at this position to allow adjustment of the tissue. Next, inject 1 μL at a rate of 0.2 L/min.

30. Move injector to the position DV -3.0 and wait 5 minutes to allow spread of the toxin.

31. Slowly retract the needle, allow about one minute to entirely remove it from the brain. Once fully out, immediately test the system by ejecting a drop of 6-OHDA solution.

32. Move to the other hemisphere and repeat the injection procedure (steps 27 to 31).

33. Clean the skull and the wound with Jodopax. Use the tissue forceps to bring the skin together and apply a thin film of tissue adhesive to the wound edges. Ensure the adhesive is evenly distributed over the wound and avoid seepage into the wound as well as eye contact. Alternatively, stitching can alternatively be used.

34. Once the wound has been closed, loosen the right ear bar, and carefully move the mouse from the stereotaxic frame to the recovery cage.

35. Inject the mouse subcutaneously with 10 mL/kg of 5% sterile glucose solution to support hydration.

36. The animal should be continuously monitored until upright posture is recovered before being returned to the home cage**.**
